# Sugar-Sick Yet Healthy: Changing Concepts of Disease in the Dutch Diabetics Association (1945–1970)

**DOI:** 10.1093/shm/hkac073

**Published:** 2023-09-23

**Authors:** Floor Haalboom

**Affiliations:** Department of Medical Ethics, Philosophy and History of Medicine, Erasmus MC University Medical Center Rotterdam, Dr. Molewaterplein 40, 3015 GD Rotterdam, The Netherlands

**Keywords:** diabetes mellitus, organised patients, disease, health, twentieth century

## Abstract

Using the journal of the Dutch Diabetics Association (*Nederlandse Vereniging van Suikerzieken*), the article provides insight into the role of an early patient organisation in conceptualising the chronic disease diabetes and its management in the Netherlands between 1945 and 1970. The dual aims of discipline (steered by health professionals) and independence (steered by diabetics) were reconciled through the concept of balance during the 1940s and 1950s. Organised diabetics played a particularly large role, and independence got particular emphasis as a consequence. This made it possible for organised patients to reconfigure their disease and identity in terms of social health in relation to labour, family and society in the post-war reconstruction period. In the late 1960s, this social concept transformed into a personal concept of health in which the concept of balance lost its prominence, despite a short intermezzo of medicalisation in the early 1960s.

On 19 November 1955, the Dutch Diabetics Association (*Nederlandse Vereniging van Suikerzieken*, NVS) celebrated its 10 year anniversary with more than 400 diabetics, diabetes experts and officials who had gathered in Amsterdam for the occasion. NVS chairman and engineer with diabetes A.J. Mollinger emphasised one aim in particular during his opening speech in the town hall:

In the past, the diabetic was lonely facing the outside world. He was a marked man, who did not qualify at all for most jobs and was given a minority complex. … It is slowly becoming common knowledge, that the diabetic, who knows to keep his balance, is an able-bodied worker. … Of course, this is only true for the well balanced diabetics. One of the main tasks of the NVS, is to give all the education and help that will result in balanced, well-regulated diabetics.^2^

In this quote, Mollinger stressed the importance of ‘balance’ (*evenwicht*) no less than three times. When diabetics succeeded in being balanced, they could contribute fully to the reconstruction of Dutch society after the Second World War, despite their disease.

Indeed, balance was central to how diabetes was framed and managed in twentieth-century North America and Europe.^3^ Physiology was an important element:

Clinical and lay texts framed diabetes as a disease of physiological imbalance, a metabolic disturbance caused by an insufficient supply of endogenous insulin, and marked by biochemical deviations, most notably elevated blood sugar (hyperglycaemia) and, in the worst cases, acid bodies (ketones) in the blood.^4^

Restoring physiological balance was the core aim of diabetes treatment, initially through diet, and after 1921 also through insulin therapy in combination with diet. Insulin therapy changed diabetes from a deadly disease for children and many adults into a chronic disease.^5^ A medical distinction between ‘severe’ diabetes with onset in young age and ‘mild’ diabetes with onset in middle age was made from the 1930s onwards, evolving into type I and II diabetes we know today in the late 1970s.^6^ In the new diabetes management programmes of famous diabetes specialists like Joslin in the USA and Lawrence in the UK, diabetes complications like blindness or cardiovascular disease became ‘less signs of diabetes than of the failure to control it’.^7^

Controlling diet, insulin and physical activities became central in these balance-aimed programmes that encompassed patients’ entire lives.^8^ As a consequence, diabetes management involved a larger role for patients, and growing interdependence between patients and their doctors. Feudtner uses Joslin’s patient files to show how the introduction of insulin transformed the illness experience of American diabetics. Patients became *morally* responsible for preventing disease complications by strictly following Joslin’s lifestyle rules, a strict diet in particular. Falk argues that in this process, a new type of patient came into being, who was essential for the doctor’s knowledge collection about diabetes.^9^ Moore argues that the personal and social lives of patients became more influential in managing diabetes in British specialist out-patient clinics.^10^

Against this background, balance became central in twentieth-century cultural and political conceptualisations of the ‘good diabetic’. The ‘good diabetic’ was someone who achieved balance not only in a physiological but also in emotional and psychological senses.^11^ Tuchman demonstrates that such understandings of who was a ‘good diabetic’ and who was not were not only gendered, but also racialised throughout the twentieth century.^12^ In the USA and Western Germany for example, white and middle-class ‘good diabetics’ were even presented as models for citizens without diabetes.^13^

Diabetic balance was not restricted to physiology in the Netherlands either, as was already evident in the jubilee argument of the Dutch Diabetics Association chairman that a balanced, male diabetic could work. Indeed, national diabetics associations founded in Europe and North America in the mid-twentieth century played an important role in the discourses on diabetes management.^14^ Moore analyses the British Diabetics Association of the early 1930s as an important source for reconfiguring the diabetic identity in terms of balance.^15^ Prüll and Harper also show how the German Diabetics Association and New Zealand diabetics societies played a major role in reshaping diabetic identity, with a large role for organised diabetics.^16^

These associations are therefore examples of the rise of specific disease organisations before the 1960s and 1970s patient organisations.^17^ Diabetics associations were founded by medical experts *and* patients, not just patients, and did not set out with the same political and social health activism that would characterise the patient organisations of later decades. Their founding occurred in an earlier period in which the authority of the doctor was not yet fundamentally challenged, but patients did claim a role in these associations. The diabetics associations are therefore early loci in which a more interdependent relation between medical experts and patients was configured, while simultaneously the emancipation and empowerment of diabetics in society was sought. Studying these associations can help us to understand changes in how diabetes was conceptualised by medical experts and patients who had become more dependent on one another in managing this chronic disease not only in a clinical context but in daily life.^18^

This article studies the role of another western European diabetics organisation in conceptualising diabetes during the first decades of its existence: the Dutch Diabetics Association (*Nederlandse Vereniging van Suikerzieken*, NVS). How did organised diabetics and health care professionals conceptualise diabetes, its management and the ‘good diabetic’ in the Netherlands? What meaning did balance get, how did this change over time, and how did this differ from other national contexts? To answer these questions, I have analysed the journal of the NVS between 1945 and 1970.^19^

The case of the Dutch Diabetics Association warrants historiographical attention for two reasons. First, patients had a relatively large role compared to other national diabetics associations. The NVS positioned itself explicitly as an ‘association of sugar sick themselves’^20^ led by diabetics rather than medical experts. This was different from the doctor-controlled American Diabetes Association and health professional-dominated British Diabetic Association.^21^ The Dutch organisational set-up was comparable to the German Diabetics Association (*Deutsche Diabetikerbund*, founded in 1951) and the Diabetic Association of New Zealand (founded in 1962), with official leading roles for diabetics and advisory roles for medical doctors. The Dutch NVS journal provides better insight in the perspective of organised diabetics than the German one, because the Dutch editorial board was less dominated by medical doctors. Second, the Netherlands lacked ‘a dominant figure’ like leading medical diabetes specialist Joslin in the USA or Lawrence in the UK.^22^ As a consequence of the decentralised, privately organised ‘patchwork’ healthcare system,^23^ a more diverse network of healthcare professionals (including doctors, nurses and dieticians) and organised diabetics steered diabetes discourse.

This article shows how Dutch organised diabetics and healthcare professionals gave diabetes management and identity new meanings in the mid-twentieth century NVS. The somewhat conflicting dual aims of diabetic control and independence were reconciled through the concept of balance. This made it possible for organised patients to reconfigure their disease and identity in terms of health. What is particularly striking about the NVS discourse compared to other national discourses in the same period, is the strong emphasis on independence. The article therefore provides new insight in organised patient perspectives on diabetes and illustrates how conceptualisations of diabetes and diabetic identity were context-specific. In part one, I show the emergence of a social concept of health during the 1940s and 1950s. In part two, this social concept transformed into a personal concept of health in the late 1960s, despite a short intermezzo of medicalisation in the early 1960s. In this shift, the concept of balance became less important.

Important in this regard is how I have translated Dutch words for diabetes and diabetics into English, because these phrases were at the heart of debates about diabetes and diabetic identity in the NVS between 1945 and 1970. The name organised diabetics initially chose for their association, the *Nederlandse Vereniging van Suikerzieken*, literally translates as ‘Dutch Association of Sugar Sick’, because the Dutch colloquial phrase for diabetes is ‘sugar disease’ (*suikerziekte*). This was inflected into the adjective ‘sugar-sick’ (*suikerziek*) meaning diabetic, and into the nouns ‘sugar-sick person’ (*suikerzieke*) or ‘sugar patient’ (*suikerpatiënt*) meaning diabetic. I use the nineteenth-century English terms as translations in order to be able to analyse historical actors’ distinction between these words and the more formal ‘diabetes’ (*diabetes*) and ‘diabetics’ (*diabeten/ diabetici*). In 1972, organised diabetics changed the name ‘Dutch Association of Sugar Sick’ (*Nederlandse Vereniging van Suikerzieken*) into ‘Dutch Diabetes Association’ (*Nederlandse Diabetes Vereniging*). We will find out what these changes in diabetes language had to do with balancing diabetes.

## Balancing Discipline and Independence to Reach Social Health in the Post-war Dutch Diabetics Association (1945–1960)

The decentralised organisation of the NVS and the involvement of both diabetics and different healthcare professionals would shape conceptualisations of diabetes in terms of balance and social health in the reconstruction period. Several diabetics and medical doctors founded the Dutch Diabetics Association (NVS) immediately after the Second World War in 1945. As was the case in Belgium, Sweden and New Zealand, the shortage of insulin during the war was the immediate incentive,^24^ but it was also a realisation of pre-war plans. The role of patients and medical doctors differed in these different national diabetes associations. For example, medical doctors founded the American Diabetes Association in 1940 as an association for experts only: patients were not allowed as members until 1970. Diabetics could become members of the British Diabetic Association, but healthcare professionals dominated in leadership and membership.^25^ In the Netherlands and Germany on the other hand, the diabetes associations were ‘the initiative of the sugar sick themselves’ from the beginning.^26^ To stress this emancipatory aim, the founders included the Dutch colloquial word for diabetics, ‘sugar sick’ (*suikerzieken*), in the name.^27^ To support the involvement of as many diabetics as possible, the NVS was set up as an umbrella organisation of local departments.^28^

As a new civil organisation, the NVS fitted Dutch political culture of the post-war period.^29^ The NVS was founded at a time in which the large role of civil society in the Netherlands was both continued and rethought. Instead of a strong centralised government, a large variety of civil organisations based on different ideological and religious outlooks for every realm of social, economic and cultural life existed. Healthcare too was a decentralised, privately and locally organised ‘patchwork’ system. As confessional parties had a large say in Dutch coalition governments, the post-war welfare state got the shape of the government supporting this existing decentralised healthcare system rather than replacing it as happened in the UK National Health Service. After the war, the argument to overcome such so-called ‘pillarisation’ of Dutch society gained wide support, but simultaneously ‘pillarised’ organisations continued to shape the political culture until deep in the 1960s. The NVS was another civil organisation with a strong emphasis on local organisation, but it was also founded as a neutral organisation in which different religious and political perspectives could co-exist.

Because diabetics played a large role in the NVS, its journal is a valuable source to study how particular types of patients and health care professionals interdependently conceptualised diabetes in the mid-twentieth century. The NVS was led by diabetics from the upper-middle class: its director was a diabetic, and the general board of the association was elected from NVS members–mainly diabetics and parents of diabetic children.^30^ Different from the USA and Great Britain, the Netherlands lacked a dominant, leading diabetes specialist like Joslin or Lawrence. Instead, a variety of healthcare professionals, including doctors, nurses and dieticians, were involved. Like the German Association, the NVS had a Medical Advisory Board to ensure that medical and dietary expertise informed the Association’s policy.^31^ One member of this Medical Advisory Board, a doctor specialised in diabetes, was medical advisor for the NVS and had a relatively large role in the Association’s leadership and on the editorial board of the NVS journal. But the editorial board consisted of both diabetics and healthcare professionals, and medical doctors only dominated it during the first five years of its existence.^32^ The local departments were led by diabetics and experts too, with their own journal section ‘Departments news’.

This new interdependence between diabetics and different healthcare professionals also resulted in tension. In this period, the attitude of medical doctors was generally paternalistic, and many were initially ‘somewhat suspicious’ of such a central role for patients.^33^ One member of the founding board, J.M. Gurck-van Vollenhoven, ‘was convinced as a doctor, that this association of lay people would make the biggest blunders without all-round medical education’.^34^ According to the first medical advisor of the NVS, F. Gerritzen, at stake was ‘difficult territory, namely the boundary for the lay person, in his pursuit of better treatment or in his attempts to offer help to doctors who are treating diabetics’.^35^ On the other hand, the socialist chairman of the Amsterdam NVS department S. Lissauer in particular argued lay diabetics should have the lead.^36^

To make sure ‘that lay people do not infringe upon medical territory’,^37^ organised diabetics and healthcare professionals created a major task division between ‘medical’ and ‘social’ aspects of diabetes. ‘Lay’ NVS members - diabetics and family members of diabetics—got a say over the social aspects, and ‘medical experts’ over the medical aspects of diabetes. But friction about this task division, the exact boundary between social and medical aspects of diabetes, and which aspects should be prioritised continued to spark debate during the first decades of the NVS’ existence.

Largely initiated by the Dutch NVS, the distinction between medical and patient responsibilities was used internationally too.^38^ The International Diabetes Federation was founded in September 1950 to bring different national diabetes associations together, and published its own journal *News Bulletin*. In line with a more general interwar ideal of science internationalisation in the Netherlands,^39^ the NVS prioritised the IDF, and its influence on the IDF was relatively large. The IDF was founded in Amsterdam, its first conference was held in the Netherlands in 1952, and NVS leaders occupied formal IDF positions. This Dutch influence extended to the task division between diabetics and healthcare professionals, although a large role for patients was also controversial in the IDF.^40^ According to NVS medical advisor and IDF board member Gerritzen, it was ‘maybe for the first time in history, that patients and doctors discussed the shared problems’.^41^ Although this quote suggests that patients and doctors had ‘shared’ discussions too, this was only true to a certain extent. A board of diabetes specialists was accountable to the IDF Council consisting of medical and *‘*lay’ representatives of every national diabetes association.^42^ However, at the IDF’s international diabetes congresses, medics discussed scientific diabetes developments, and diabetics discussed ‘social and personal problems’.^43^

In the NVS, ‘medical problems’ concerned everything that was associated with medical expertise about diabetes as ‘a disorder … of the sugar metabolism’, that could not be cured, but could be treated with diet, insulin and physical exercise.^44^ These medical problems were the province of experts in the NVS: doctors in the first place, but also nurses, and food scientists and dieticians affiliated to the government Nutrition Education Bureau (*Voorlichtingsbureau voor de Voeding*).^45^

Both diabetics and healthcare professionals were concerned with the ‘social problems’ of diabetes, but key NVS members framed these as diabetics’ realm in particular.^46^ These social problems concerned a broad range of individual and collective problems in the post-war reconstruction society, like access to the labour and insurance market, but also diabetes care, collaboration between diabetes associations and the government, and diabetes statistics. Primarily based on their specific social problems, the NVS journal categorised diabetics into diabetic children, young diabetics, working diabetics (including housewives) and the diabetic elderly in the 1950s.^47^ Similarly, a distinction between diabetes with and without complications was also made primarily in relation to the social issues of labour and insurance.^48^ Only classification of diabetics on the basis of low and high body weight was primarily discussed as medically rather than socially relevant.^49^

As a result, different people with different backgrounds shaped the contents of the NVS journal: diabetics, dieticians, nurses, doctors and the occasional ‘not “learned” …. [P]erfectly common person’.^50^ Their distinction between medical and social aspects of diabetes related to two well-known themes in diabetes historiography: discipline and independence.

## Healthy Diabetics: Balancing Discipline and Independence

In 1947, a Frisian diabetic wrote the NVS indignantly that hospital observation had *made* him ill:

I consider it incorrect that, during check-up in a hospital, one is treated as a sick person, while, except for the sugar [diabetes], one is completely healthy. One must stay in bed, which makes an otherwise healthy person ill.^51^

Indeed, in the NVS post-war discourse on diabetes, conceptualisations of health were closely related to conceptualisations of disease, and of equal significance. The concept of ‘balance’ was central in this discourse on diabetic health, which was defined in *social* ways in the post-war reconstruction period.

‘Balancing’ ideals of discipline and independence was central to modern diabetes management and discourse in different European and North-American countries: daily management of the disease came with strong normative goals about being in control and being responsible for a disciplined life as a diabetic.^52^ This included a paradox: diabetics needed to become independent *by* strictly following medical prescriptions. For historical actors, this tension was solved through the concept of balance. The precise meaning of balancing discipline and independence differed in various places.

In the Netherlands, these meanings were shaped by a more general political culture in which values of independence were combined with the social ties and disciplined working ethos of the post-war reconstruction period.^53^ Independence got particular emphasis: it was such a central political value in this period, that Van Klaveren has called his cultural history of post-war Dutch healthcare ‘The independence syndrome’.^54^

In the NVS discourse, the ‘balance’ *(evenwicht*) between discipline and independence was related to the distinction between medical and social aspects of diabetes discussed above. In the first place, the central importance of discipline in conceptualisations of diabetes was strongly connected to the medical expert perspective. Historical actors did not use the word ‘discipline’ itself: I use it as an analytical category to group words like ‘control’ (*controle*), ‘orders’ (*voorschriften*), ‘life rules’ (*leefregels*), ‘regularity’ (*regelmaat*), ‘moderation’ (*matigheid*) and ‘self control’ (*zelfbeheersing*). Medical experts set the rules that patients needed to follow with regard to the strict medical triad of diet, insulin and physical exercise.^55^ Diabetics, doctors, dieticians and nurses agreed on the importance of ‘medical control’ and ‘the doctor’s orders’.^56^ According to the director of the NVS and diabetic Bloemendaal there was ‘only one sound method … for diabetics: stick to the diet and inject insulin if necessary, everything in close consultation with the treating doctor’.^57^ Patients were expected not to adjust such orders.

The second core concept, independence, was particularly important in the Dutch NVS, as it was in Dutch healthcare and society more generally. It summarised the emancipatory answer to the ‘social problems in relation to diabetes’, which was conceptualised as the domain of diabetics themselves.^58^ Historical actors used words like ‘independent’ (*onafhankelijk*/ *zelfstandig*), ‘stand on one’s own feet’ (*op eigen benen staan*), ‘self-help’ (*zelfwerkzaamheid*), ‘look after oneself’ (*zichzelf helpen*), ‘ordinary/ normal people’ (*gewone/ normale mensen*) and ‘a full member of society’ (*volwaardig mens/ lid van de maatschappij*). Independence was understood as being able to function in society independently of one’s social circle, a goal for which the creation of the welfare state was increasingly accepted as a means.^59^ Simultaneously, civil organisations like the NVS continued to play a large role in this ideal too. In comparison to diabetes associations abroad, the NVS positioned itself proudly as a ‘lay organisation, the organisation of self-help’.^60^

When patients dealt with their diabetes in a ‘balanced’ (*evenwichtig*^61^), ‘responsible’ (*verantwoordelijk)* and ‘sensible’ (*verstandig*) way, they made it possible to free themselves from being ‘ill’ (*ziek*) and ‘dependent’ (*afhankelijk*), and to reach a state of health. Citing the British *The Diabetic Journal*, Lissauer for example emphasised:

the diabetic [should] strive for maintaining the most complete good health as possible by the greatest possible degree of common sense, without turning this pursuit into an obsession. It is not always easy to find the golden mean between pampering oneself and neglecting oneself. Yet the secret how one can live happily and successfully *with* his diabetes lies precisely here.^62^

This ‘most complete good health’ meant functioning ‘normally’ in a social sense: organised diabetics conceptualised diabetes more as ‘sickness’ in a societal sense than as ‘illness’ in a personal sense. During the 10 year jubilee in 1955, NVS chairman and diabetic J.A. Martijn argued:

Let us be aware, that in the first place we are people who need to participate in normal society as well as possible. And only then we are allowed to think of our sugar disease.

After all, we are father, mother, son or daughter, employer, employee, citizen, community member, friend etc. etc. We all have the same good and bad characteristics as other people. In short, we carry the same responsibility as the non-diabetic towards others.^63^

Indeed, this was a social mission. In 1956, NVS director Bloemendaal called on young diabetics not ‘to sit in a lazy chair at home with the slippers on’: ‘We all have a mission in society’.^64^

This discourse on diabetic health increasingly distanced diabetics from the word ‘disease’. In the 1940s, the Dutch word *suikerzieke* (‘sugar-sick person’) in the name of the NVS signified its emancipatory aims. But in the 1950s, both diabetics and experts started to argue that this word was wrong:^65^ ‘a sugar patient is not a sick person. Therefore do not treat him as a sick person, he is a completely normal person with a minor abnormality’.^66^ Doctors and nurses deliberately did not wear uniforms in the NVS children’s home in order to prevent associations with disease.^67^ A new swimming pool was welcomed as the ultimate proof of the children’s health: ‘Long let our water flow in and out of our pool! For the benefit of our unsick, sugar sick children!’^68^

How balancing medical discipline and social independence were interlinked in a social concept of diabetic health, becomes clear from a key jubilee article on ‘self-help’ by the prominent Amsterdam NVS leader Lissauer:

Well, the foundation of our N.V.S. was and is self-help. That is why it was so right that … we changed the old name “Dutch Association for the protection of the interests of sugar sick” into “Dutch Association of Sugar Sick” [in 1948]. With this, we wanted to express that we, diabetics *themselves*, should do everything that has to be done for the diabetics. Only for clear-cut medical matters, we have secured the invaluable help and cooperation of the doctors united in our Medical Advisory Board. For unlike e.g. rheumatism patients, TB patients or blind people, who *have to* call in the help of others due to their physical abnormalities and difficulties, we, diabetics, have always told our fellow diabetics and the non-diabetic world that we can fend for ourselves. Simply because we do not want to be seen as sick at all, despite the nasty name “sugar sick”, but as healthy and able-bodied people, who only have a metabolic abnormality.^69^

Healthy diabetics could balance discipline and independence in order to function normally in society.

In the Dutch post-war period, collaborative ‘education’ (*voorlichting*) was widely seen as an important means to reach this goal. Different from the British Diabetic Association’s focus on promoting specialist outpatient clinics,^70^ the NVS focused on educating diabetics in balancing discipline and independence in order to reach health. Organised diabetics, doctors, nurses and dieticians of the government Nutrition Education Bureau collaboratively educated NVS members on a national level via the NVS journal, media and meetings. Locally, many department meetings were devoted to such collaborative education too: evening programs included lectures by doctors on medical discipline, dieticians on diet and NVS leaders on the importance of an independent diabetic attitude and social issues with diabetes. Often, films were screened.

**Fig. 1:**
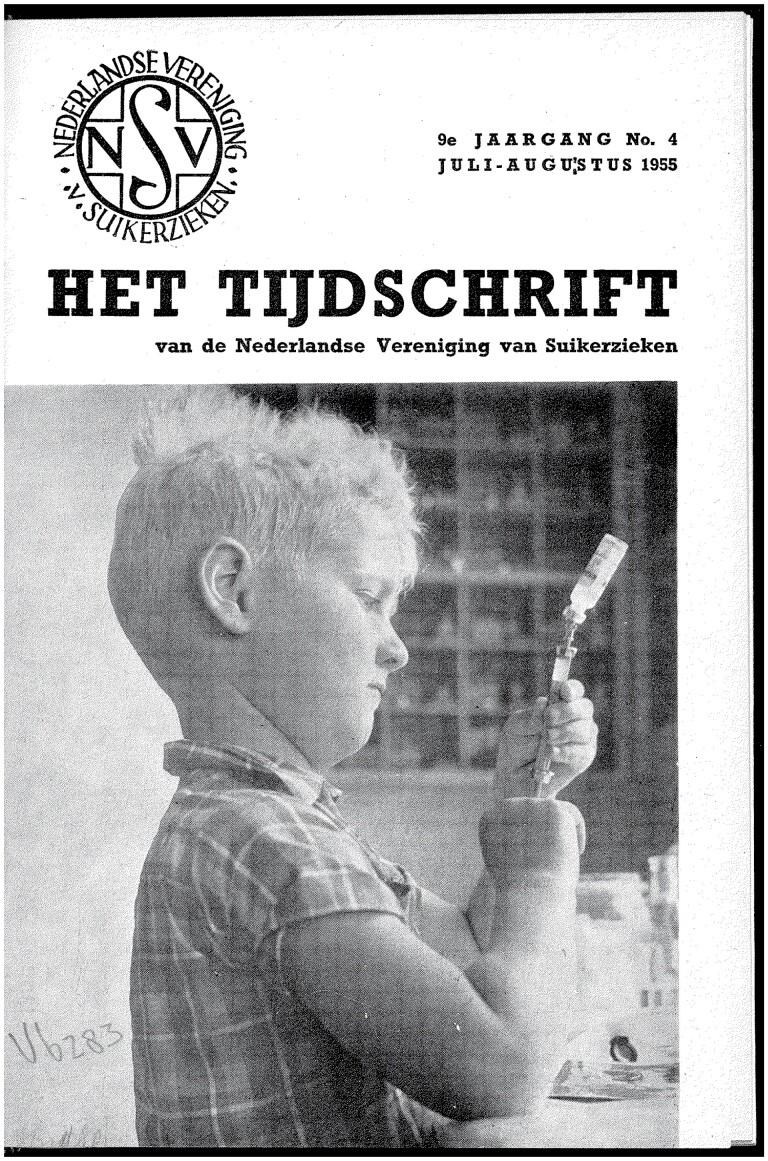
Cover NVS journal in the summer of 1955 with a participant of the NVS holiday camp. Reproduced from *Het Tijdschrift van de Nederlandse Vereniging van Suikerzieken,* 1955, *9:4*, with permission from Diabetesvereniging Nederland.

**Fig. 2:**
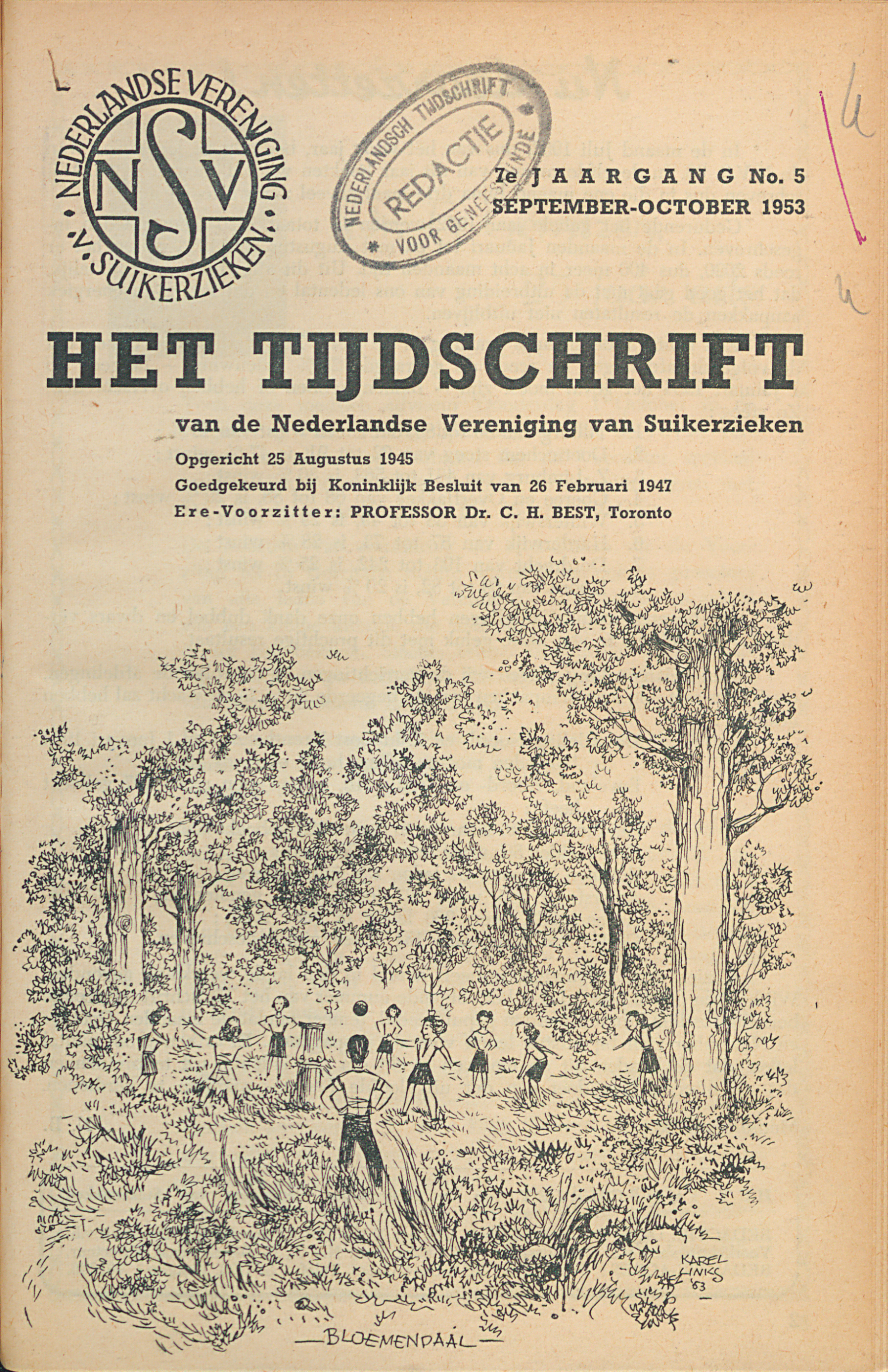
Cover of the NVS journal in the autumn of 1953, showing children playing ‘normally’ during the NVS holiday camp. Drawing by Karel Links. Reproduced from *Het Tijdschrift van de Nederlandse Vereniging van Suikerzieken*, 1953, *7:5*, with permission from Diabetesvereniging Nederland.

Central to the education activities in these early decades of the NVS’ existence were the child holiday camps—after American examples—were children with diabetes for example learned to inject themselves with insulin under medical supervision *and* played ‘normally’ in order to ‘increase their sense of independence’ (Figures 1 and 2).^71^ These camps stood in a tradition of ‘health colonies’ for children. In the late nineteenth century, teachers originally founded holiday colonies for poor and undernourished children, like happened in other European countries. In the 1920s, the Dutch medical profession had transformed these camps into therapeutic places for ‘weak’ children to prevent disease with sunshine and fresh air, tuberculosis in particular. When tuberculosis became less of a problem, the health colonies shifted their focus to mental hygiene. In the 1950s, 25 private societies still ran such health colonies for children.^72^ The emphasis on a somatic disease and the combination of discipline with independence was characteristic of the 1950s diabetes holiday camps.

**Fig. 3:**
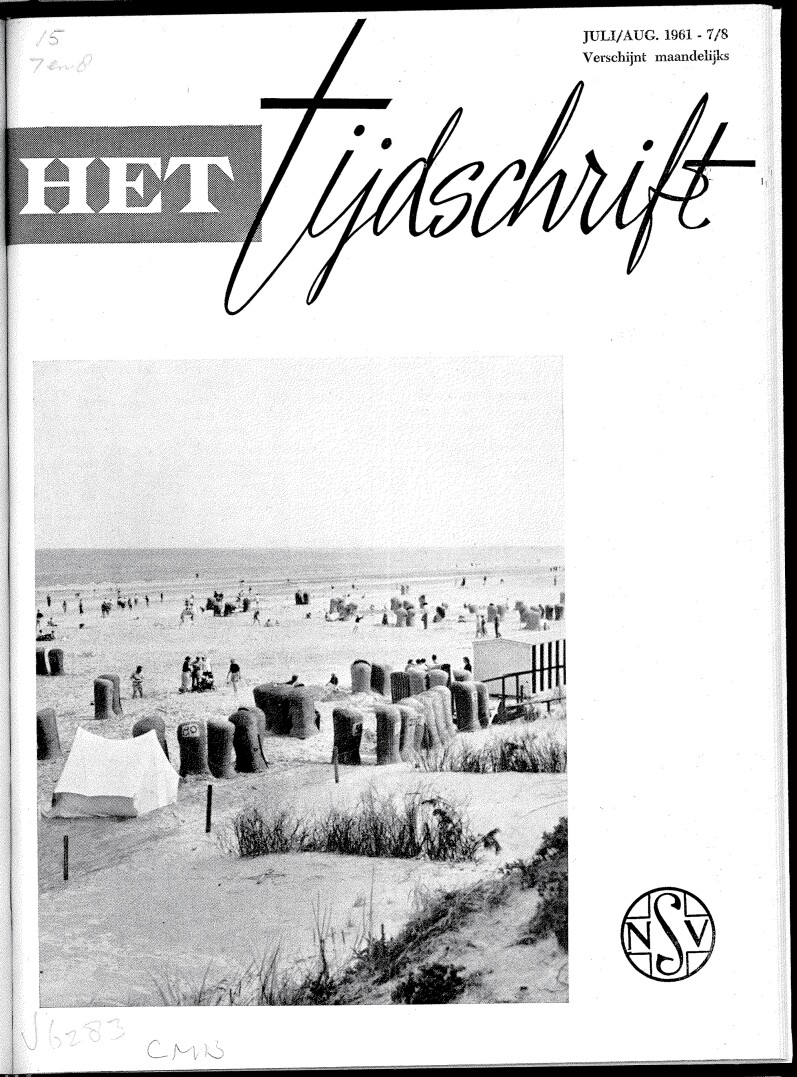
Cover of the NVS journal in the summer of 1961, showing a ‘normal’ family holiday scene. Reproduced from *Het Tijdschrift [van de Nederlandse Vereniging van Suikerzieken]*, 1961, *15:7/8*, with permission from Diabetesvereniging Nederland.

This was true for all NVS education activities: the content did not only concern the treatment triad of diet, insulin and physical exercise (discipline), but life as a whole (independence). For example, the popular 1955 NVS film about the holiday camp for diabetic children got the telling title *We go out… and act normally*,^73^ and varieties of the phrase became popular to designate that diabetics lived ‘normal’, middle-class lives, including going on holidays.^74^ Achievements in sports were another important trope to prove diabetics’ health. The covers of the NVS journal underscored this with illustrations of playing children (Figure 2), Dutch landscapes and family holiday scenes (Figure 3).

The United States, Canada and the United Kingdom functioned as central ‘reference cultures’ in the NVS education activities: cultures that had an ambivalent position by serving as key examples that were criticised at the same time.^75^ Especially the foreign leading diabetes authorities were used in such dual ways. On the one hand, for example, Dutch organised diabetics used American diabetes specialist Joslin’s late 1940s system of medals to designate who were ‘good diabetics’.^76^ In 1954, one active NVS member of the Rotterdam department won Joslin’s Victory Medal as ‘a shining example for all of us’, because he showed ‘no change at all in the body and has been able to live like a normal human being, thanks to the fact that he completely kept to the prescriptions’.^77^ On the other hand, NVS leader of the Amsterdam department Lissauer combined admiration with criticism of such leading medical authorities, for example when he visited British diabetes specialist Lawrence at Harleystreet, London: ‘All these [Harleystreet] doctors are known for their broad knowledge and high bills’.^78^ Medical expertise was not thought to be sufficient in the NVS journal: the right diabetic attitude was crucial for succeeding in balancing discipline (steered by medical authority) and independence (steered by the right diabetic attitude).^79^ That the two did not necessarily go together was proven by ‘doctors, … dentists, … analysts’ with diabetes who ‘brushed their responsibility aside’ while they ‘actually should know better’.^80^

Education activities also addressed problems with balancing discipline and independence. Diabetes discipline was by definition ‘An unpredictable factor’ for experts: there was always the fear that diabetics would *not* follow medical orders.^81^ Different groups of professionals responded differently to this problem. Medical doctors and dieticians in particular debated the extent of diet discipline that was needed and feasible, and this included disagreements ‘about the place of the dietician in diabetes treatment’.^82^ From the 1930s onwards diabetes specialists had debated the question of whether the diabetes diet needed to be strict or ‘free’.^83^ From the start of the collaboration between the NVS and the Dutch Nutrition Education Bureau, the ideas about the diabetes diet in the NVS were relatively liberal, be it not totally ‘free’—this type of diet was referred to as ‘regulated diet’.^84^ More than doctors, dieticians paid attention to the difficulties of sticking to the diet in their education activities. Government dietician Diane ten Haaf had a large role in the early years of the NVS, both in the journal and the many local education meetings. She expressed understanding for diabetics’ ‘so human reaction’ of dodging the rules, and thought it crucial to consider personal and social circumstances of diabetics.^85^ Advocating for a certain extent of flexibility in diet, she preferred the word ‘diet suggestions’ (*dieetwenken*) rather than ‘diet prescriptions’ (*dieetvoorschriften*^86^) given by the doctor.^87^ In later years, experts of the government Nutrition Education Bureau continued to emphasise the importance of diet variation, and developed an extensive system of ‘equivalents lists’ together with the NVS Medical Advisory Board to enable diabetics to substitute food items. In their view, the diet for diabetics with a ‘normal weight’ was almost similar to a ‘normal diet’.^88^

Organised diabetics discussed both the societal and the self-image of people with diabetes as interlinked threats to their essential independence in education activities. Mollinger’s ‘minority complex’ cited in the beginning of this article was a general issue of concern.^89^ According to NVS director and diabetic Bloemendaal, 80 to 90 per cent of the diabetic children who joined the NVS holiday camp were ‘very spoilt’ by their caregivers (their mothers in particular) with the likely result of a similar ‘minority complex’.^90^ Spoiling was therefore a no-go: ‘Sugar ill children … should not be treated as cracked eggs’.^91^ The same was true for ‘spoiled’ adults with diabetes, who became ‘difficult diabetes patients’ and ended up ‘more or less terroris[ing their] environment’.^92^ The ‘“pity”’ of others^93^ and diabetic ‘self-pity’^94^ were ‘very undesirable!’ in ‘our fight to get the sugar sick acknowledged as a full member of society’.^95^ Society at large, family members and diabetics themselves all needed to be educated to change their understanding of diabetes.

As happened elsewhere, models of the ‘good diabetic’ often occurred in this NVS education discourse on balance. In some instances, diabetics were even discussed as being *healthier* than non-diabetics. As Tuchman shows in her history of racialised diabetes, diabetes was considered a ‘disease of civilization’ in the US discourse of the mid-twentieth century, and white, middle class diabetics who managed their disease well were seen as model citizens.^96^ In the NVS journal too, disciplined and independent diabetics were presented as model citizens.^97^ In 1961, Mrs Rutgers-Mees wrote to the NVS journal to commemorate her late husband who was extraordinary healthy: ‘Despite his “sugar” he did his job excellently, with great cheerfulness and vitality, and he did his gymnastic exercises every morning on the balcony, humming to the beat’.^98^

As happened in British and American diabetes discourse,^99^ Othering played an important role in clarifying how a diabetic could fail to reach balance and health. Being of colour, female or poor was considered to make it less likely that the patient could reach the necessary independence through discipline. Two ‘wrong paths’ were a sign of failure to balance: not following the rules, or adopting a too dependent attitude. According to one doctor at a local NVS department meeting, among the ‘many liars and sinners’ among his diabetes patients, ‘[t]he weak sex in particular’ had ‘the nasty characteristic to continuously insist not to have sinned’.^100^ Discussing the ‘diabetes problem’ abroad, Lissauer argued that some patients followed dietary rules *too* strictly: ‘Moreover, the native Moroccan is such a simple soul in general and so impressionable as a consequence, that when he accepts the diabetes diet, he carries it through to such an extent that it leads to malnutrition’.^101^ Comparisons between the Netherlands and its former colonies were similarly used to contrast white, middle-class model diabetics with less successful Others.^102^

In short: the emphasis on discipline and independence came with a strong moral message on how an ideal diabetic should balance these values. This is in line with historians of diabetes’ analyses of the moral dimensions in diabetes discourse in this period. In the Netherlands, organised diabetics themselves played a major role in spreading this moral message, and in their perspective social dimensions of diabetes were of central importance. These ideals fitted in the post-war reconstruction period and political culture: a good diabetic lived a normal family, working and citizen’s life, and learned about how to do this through education organised collaboratively by civil society, healthcare professionals and the government. Indeed, moral ideas about how to balance diabetes were strongly related to conceptualisations of health—not as the opposite of disease, but as inherently part of living with a chronic illness.

The particular emphasis on the social independence of ‘healthy’ diabetics, shows that autonomy of diabetics meant something very different in the post-war reconstruction period than what historians identify as patient autonomy in the 1960s and 1970s.^103^ During the 1950s, being autonomous as a patient did not mean to be able to make medical decisions or be heard as experts, but to be an autonomous citizen *by* following medical rules. This was patient autonomy in a social sense, not the more individualised patient autonomy of later decades. Indeed, the emphasis on social health in the NVS discourse would change during the 1960s.

## The Rise of Personal Health in the Dutch Diabetics Association (1960–1970)

During the early 1960s, the original distinction between medical and social aspects of diabetes was blurred, and social aspects of diabetes were increasingly medicalised both internationally and in the NVS. Doctors involved in the NVS increasingly thought age groups of diabetics—diabetic children, adults and elderly people—of *medical* rather than social importance. This shift was connected to the growing popularity of antidiabetic drugs for certain patients introduced in the 1950s.^104^ In the 1960s, young, adult and elderly diabetics had become ‘three types of diabetes’ with specific characteristics and preferred treatments.^105^ Diabetic drugs were the preferred treatment for the ‘stable diabetes’ of elderly people who did not need insulin.

This rise of new diabetic drugs was part of the quick expansion of professional diabetes healthcare under the post-war welfare state, which had impact on diabetes organisations too. In the International Diabetes Federation, rising numbers of medical experts soon truly outnumbered lay diabetics.^106^ As a consequence, the themes that had formerly been referred to as ‘social’ were called ‘medical-social’ in the early 1960s. Lay NVS representative Bloemendaal complained about ‘too much … medical-scientific jargon’ and neglect of the popular NVS theme of labour and diabetes in the ‘medical-social section’ of the 1961 IDF congress in Geneva.^107^ In 1965, the social aspects of diabetes had disappeared completely from the IDF congress programme: the IDF chairman referred to them as ‘clinical questions’.^108^

In the Dutch NVS, the medicalisation of social aspects of diabetes was also the result of the individuals who shaped the content of the journal. A medical perspective was relatively dominant in the journal during the first half of the 1960s, although diabetics made up half of the editorial board of the NVS journal from 1953 onwards, and even dominated the editorial board from the end of 1963 onwards. The influential NVS leaders and lay journal editors Lissauer and Bloemendaal died in 1958 and 1962, respectively, and the new lay editors put less emphasis on the social aspects of diabetes. Doctor, NVS medical advisor and IDF secretary dr. Jac. J. Witte on the other hand medicalised the journal content with extensive medical reports. More than the first NVS medical adviser Gerritzen, he was an outspoken advocate of a paternalistic approach to diabetes management—he continuously emphasised the importance of diabetics’ discipline under the authority of medical doctors.^109^

Moreover, following the developments within the international association IDF, Witte approached the diabetes problems of diabetic children, adults and elderly people as medical rather than social problems. In his reports of the NVS holiday camps for children, for example, Witte focussed on numbers and medical control rather than the social experiences of the children that had been of central interest before.^110^ This is in line with a more general trend of medicalisation in health colonies, although it did not lead to criticism of the existence of the diabetes holiday camps, as happened with other health colonies in the Netherlands during the 1950s and 1960s.^111^ Organised diabetics’ strong support for the health camps, and the children having a somatic rather than mental disorder explain this. Similarly, Witte medicalised elderly diabetics’ social problems,^112^ proposing a special NVS Diabetes Centre, with ‘*a building for the medical aspects in particular*’ as a solution.^113^

Wittes emphasis on medical diabetes related to the question whether or not diabetics should be called sick or not. Medical experts debated the status of diabetes complications in particular: were these a consequence of imperfect discipline, or were they a treatment-independent manifestation of the disease? Prominent NVS doctors like Witte increasingly believed the latter was the case. Witte even preferred the phrase ‘accompanying symptoms’ (*begeleidende verschijnselen*) instead of ‘complications’.^114^ Sometimes, this had consequences for doctors’ perspective on the importance of lifestyle discipline. Schreuder ‘sometimes’ thought ‘it was a bit unfair … to say to a patient with so-called complications of sugar disease: it is your own fault, if only you had been more careful’.^115^ In 1961, NVS medical advisor Witte sharply disagreed with NVS members’ proposals to rename the ‘old-fashioned’ ‘Dutch Association of Sugar Sick’ as ‘Dutch Association of Diabetics’ (*Nederlandse Vereniging van Diabetici*):

When one says “the name sugar sick is old-fashioned”, one means to say that because of the possibilities of treatment a sugar-sick person does not need to feel ill anymore. … This does not change the fact that treatment remains necessary, however. Why should we replace a Dutch word [sugar sick, *suikerzieken*] with a foreign word [diabetics, *diabetici*], which means the same thing? The advantage is that a foreign word, because it is not immediately understood, is more kindly appreciated. The disadvantage is that we are actually burying our heads in the sand a little bit. The Dutch word also has the advantage that it keeps us awake, it makes us watchful. It tells us: “Well, at the moment you don’t feel ill, you can do everything, thanks to the treatment. Please comply with it, otherwise you *will* feel ill.”^116^

Soon, organised diabetics would again strongly criticise this emphasis on disease.

## The Return of the Healthy Diabetic

World-wide authority-critical perspectives on culture, politics, gender roles, religion and knowledge changed Dutch society rapidly and relatively peacefully from a conservative into a progressive nation during the 1960s.^117^ Political historian James Kennedy explains this rapid shift by emphasising continuity: the generation in power facilitated many changes demanded by the younger generation.^118^ The Dutch NVS too saw continuities with the preceding decades, as well as change as a consequence of new critical patient perspectives.^119^ A late 1960s controversy led to several seemingly sudden schisms in the association. In 1967, the Amsterdam department left the NVS and founded its own diabetes association.^120^ In 1969, both NVS medical advisor Witte and chairman Wilton resigned from their positions, and in 1970, the entire NVS Medical Advisory Board resigned. These changes did not form a total rupture, but were a continuation of the NVS’ earlier emphasis on diabetic independence and health, be it in new ways.

In the second half of the 1960s, NVS diabetics again emphasised that diabetics were healthy rather than ill, and criticised the use of the words ‘sugar disease’ (*suikerziekte*) and ‘sugar sick’ (*suikerzieken*).^121^ These criticasters preferably used ‘diabetic’ (*diabeet*) or ‘sugar patient’ (*suikerpatiënt*) to avoid the word disease (*ziekte*) or being sick or ill (*ziek*).^122^ A participant of a NVS youth weekend reflected afterwards:

A well-regulated ‘sugar-sick person’ is not sick, but has an inconvenience. … From now on, this name must go. The young people should start with that. If every [young] and older diabetic is convinced that he is a diabetic and not a sugar-sick person, we will quickly lose this name.^123^

The argument was framed as a young generation argument, but it was actually not a new argument in the history of the NVS as we have seen already. When diabetic and active NVS member A.F. Burmeister–Visser joined the editorial board in 1968, she played with the criticism of the phrase ‘sugar disease’ by calling her new journal section ‘Experiences of a healthy, sugar-sick person’.^124^ In the same year, the rebellious Amsterdam department got rid of the phrase ‘sugar sick’ (*suikerzieken*) and renamed itself ‘Dutch Diabetes Foundation’ (*Nederlandse Diabetes Stichting*).

Although the emphasis on health returned, it was a new concept of *personal* rather than social health in which the meaning of independence and discipline gradually changed too.^125^ First, the personal rather than the social became central in the ideas about diabetic independence as compared to the 1940s and 1950s. During the 1960s, independence continued to be an important ideal in Dutch society, but ‘the 1950s sense of duty made place for freedom of choice and self-fulfilment’ according to Van Klaveren.^126^ Diabetes could only be managed when the diabetic got to know the characteristics and peculiarities of her disease over time. In 1962, one diabetic argued in a letter ‘that every diabetic is different, and we diabetics can learn little from each other, unfortunately’.^127^ Soon, however, personal peculiarities and experiences gained importance as a source of knowledge and insight: ‘There can never be enough exchange of ideas about the daily life of the sugar patient’.^128^ In 1970, Burmeister–Visser explicitly explained its value:

In summary, I believe that every *new* sugar patient should know that there is a great deal to be learned about his disorder and that he can usually only learn the hard way. It can therefore be useful to hear or read many experiences of other diabetics^129^

In 1970, personal diabetes also came to the fore in the ‘new editorial direction’ of the NVS journal, which focused on the exchange of diabetics’ experiences and opinions.^130^

Organised diabetics now emphasised that independent diabetics needed to get to know their own unique diabetes in daily life, which was itself unique and personal rather than fitting social patterns. This is for example evident from the shared personal diabetes experiences in the new 1970 journal section *NVS Diabrief*—‘dia letter’, a selection of letters of NVS members. One letter writer explained that he had been ‘so far only moderately successful’ in a regular life prescribed by doctors, due to all kinds of peculiarities in ‘profession, disposition and many other factors’.^131^ He had adjusted the general medical advice to ‘my personal experience’. In another letter, a 20-year woman with diabetes reported on her successful participation in a Kennedy march (walking a distance of 80 km in 20 h) to ‘prove that if you are diabetic, you do not belong to a group of people who are sick’.^132^ She emphasised the importance of gaining self-knowledge as a personal and gradual process ‘together with your doctor’.

Indeed, ideas about discipline, and about how medical experts and lay diabetics related to one another also changed. Discipline changed from being dictated by medical orders, into an individual attitude that depended on personal experience and expertise of the diabetic in close consultation with healthcare professionals. As the previous quote by the Kennedy march walker shows, diabetics and medical experts were still thought to have an interdependent collaborative relation. New was the rejection of hierarchy in this relation. By knowing diabetes as a personal disease, diabetics matched or even surpassed medical professionals in expertise about their disease and health. A young woman with diabetes critically evaluated the educational talks by doctors and NVS board members on this point during a NVS youth weekend in 1970:

In the first place, finding out as much as possible about our ailment is a good idea of course, but this is quite easy through the monthly magazine ‘diabc’ and other literature. But only factual knowledge is not nearly enough, I think. The mutual contact, the questions in the discussions following both speakers’ introduction, were actually more interesting than the introductions themselves.^133^

Exchange of personal experiences, circumstances and peculiarities was what really mattered.

NVS diabetics’ explicit criticism of medical authority was a fundamental departure from the formerly shared perspective that medical aspects were medical terrain on which lay people had no say.^134^ In the NVS and the German Diabetic Association, this occurred more than two decades earlier than in the British Diabetic Association.^135^ According to Burmeister-Visser, ‘the non-sugar patient needs information [about daily life with diabetes] just as much as the sugar patient’.^136^ The Kennedy march walker criticised diabetes doctors for their ‘bad education’.^137^ The most telling example is several local NVS departments’ open rejection of the Medical Advisory Boards advice on diabetes screening in late 1969. NVS medical advisor Witte had compared diabetes screening of the population to ‘a medical “razzia”’,^138^ and the Medical Advisory Board advised against it. This inspired unprecedented criticism from local NVS departments.^139^

Changes in the NVS journal also reflected the new meaning of discipline and the relation between doctors and diabetics. ‘[T]he bickering’ of the late 1960s included friction about the NVS journal: ‘the criticism of the content [was] heavy’.^140^ The long and detailed medical articles written by NVS medical advisor Witte, and the lack of attention for discussion and exchange of experiences of diabetics themselves, were a source of frustration for the NVS ‘“rebels”’.^141^ Active and critical NVS members like B.H.J. Evers (Haarlem department), A.F. Burmeister-Visser (Delft department) and D.J. Endeman (Amsterdam department) joined the editorial board in 1965, 1968 and 1969 respectively, and lay editors became the majority of the editorial board. The medical influence on the content of the journal diminished. New sections were introduced on the perspective of diabetics, their daily lives and debate about patient experiences.

As a consequence of this rise of the personal in the NVS discourse, the idea of balance lost its prominence. In the UK, the British Diabetes Association renamed its journal *Balance* in 1961.^142^ Nothing of the sort happened in the Netherlands.^143^ Indeed, during the 1960s, independence and discipline had in some ways collapsed into each other, and the need to *balance* the two had lost its urgency. The idea that the good diabetic was a ‘model citizen’ also became less prominent in the discourse focussed on personal peculiarities.

But the idea that diabetics were healthy did not lose its prominence, and the question whether diabetes should be conceptualised as a disease or not became the major source of conflict in the late 1960s. During the general meeting of the NVS in 1968, NVS medical advisor Witte used East-German diabetes specialist Katsch’ phrase of conditional health to explicitly reject the return of the healthy diabetic in the NVS discourse:

Healthy sugar sick do not exist; conditionally healthy do. … However, it is not the case that – if one sticks to the rules – one is sure not to get any complications. Sugar disease is a very serious disease, about which we know a lot, but still too little to be able to cure the disease.^144^

This use of ‘conditional health’ was a little ironic, because Katsch had used it to argue that diabetics were ‘able to work’.^145^ Some NVS leaders were convinced by Witte’s conclusion that doctors should be setting the rules, and patients should follow.^146^ NVS chairman and diabetic Wilton considered his initial idea that diabetics could live ‘as normal healthy people’ as ‘a huge mistake’ in 1968: ‘Because it is not true. We do a group of sugar patients great injustice if we try to convince them that sugar disease is not so bad’.^147^ For Wilton too, this implied that medical doctors should have a leading role in both the doctor–patient relationship and in the leadership of the NVS.

Instead, the authority of medical doctors was no longer self-evidently accepted in the NVS. This was too much for many medical experts involved in the NVS: Witte resigned as medical advisor in February 1969,^148^ and the entire Medical Advisory Board quitted in February 1970.^149^ As a consequence, medical influence on the journal content diminished. Between the departure of Witte in February 1969, and the temporary return of former medical advisor Gerritzen in July 1970, the editorial board of the NVS journal had no medical member at all.^150^ The new Medical Advisory Board in 1970 was no longer dominated by medical doctors, but had ‘a broader base than was previously the case’.^151^ This included the return of a dietician, and representatives from fields concerned with social problems of diabetes, like the Directorate General of Labour, the Chief Public Health Inspectorate, the Dutch Organisation for applied science (TNO), the Central Bureau for Driver Licenses (CBR) and an insurance doctor.

Wittes criticism of diabetic health was generally dismissed, and NVS leaders started to support the calls to get rid of disease and illness connotations. Soon after the departure of Witte and Wilson, the Amsterdam department returned to the NVS. The ‘structure committee’ was appointed as a response to the late 1960s uproar and Amsterdam crisis, and prioritised the issue of diabetic health.^152^ In 1970, NVS director Rotte argued: ‘The name of our association, the Dutch Association of Sugar Sick, is misleading. A well-regulated sugar-sick person is by no means ill’.^153^ The name of the association was changed in 1972: from Dutch Association of Sugar Sick (*Nederlandse Vereniging van Suikerzieken*) into Dutch Diabetes Association (*Nederlandse Diabetes Vereniging*).

## Conclusion

National diabetes associations of the mid-twentieth century like the Dutch Diabetics Association (*Nederlandse Vereniging van Suikerzieken*, NVS) played a major role in rethinking diabetes and diabetic identity in terms of balance.^154^ Analysing the NVS journal between 1945 and 1970, I have studied how diabetics and health care professionals who collaborated in the NVS conceptualised diabetes, its management and the identity of diabetics. How did this differ from other national contexts, and how did this change over time?

Close reading of the NVS journal shows that in the Netherlands as elsewhere organised diabetics and healthcare professionals were thought to have an interdependent relation in managing diabetes as a chronic disease.^155^ In the NVS, they agreed on a task division between medical aspects of diabetes as the realm of doctors, and social aspects as the realm of diabetics themselves. The importance of the latter was emphasised in the choice for the colloquial name of the NVS, which literally translates as the Dutch Association of the Sugar Sick (*Suikerzieken*). The task division was integrated in the set-up of the association, its many education activities and it was to some extent copied in the International Diabetes Federation.

The concept of balance made it possible to reconfigure diabetes and diabetic identity in terms of health. During the 1940s and 1950s, a social concept of health was dominant in the NVS discourse. Two values were central: discipline in relation to the medical aspects of diabetes (the realm of healthcare professionals), and independence in relation to its social aspects (the realm of organised diabetics). The ‘good diabetic’ succeeded in balancing discipline in order to control diabetes, and independence in order not to be controlled by the disease. This enabled a ‘normal’ life in labour, family life and society of the post-war reconstruction period. As happened in North America and in the UK, the balanced, healthy diabetic was presented as a model citizen, while diabetic Others—mainly women with obesity and people of colour—functioned as negative examples. As part of this emphasis on balanced health, the colloquial phrase ‘sugar sick’ was increasingly criticised because of its connotations with disease.

As a consequence of the quick expansion of medical diabetes expertise, the discourse in the NVS journal saw a short period of medicalisation in the early 1960s. This shift was also driven by a new medical advisor, diabetes specialist Witte, who thought his role was to lead rather than support diabetics organised in the NVS. The distinction between social and medical aspects of diabetes was blurred, and more emphasis was put on disease rather than health, and discipline rather than independence. This also involved medicalisation of aspects that had been considered *social* aspects of diabetes before, like the use of age groups for medical understanding of the disease, and a more paternalistic view on the relation between doctors and diabetics. NVS leaders who were diabetics themselves supported this move away from the focus on social aspects of diabetes and a leading role for patients in the early 1960s. This is an important sign that the very outspoken preference for patient leadership, for example in the left-wing Amsterdam department, was not shared by the entire association.

But in the general atmosphere of societal change, criticism of the expansion of medical authority within the NVS did increase significantly in the course of the 1960s leading to major schisms. This was related to major changes in the meaning of discipline and independence in the NVS discourse. Discipline was reframed as steered by the diabetic and the medical professional in collaboration rather than by medical authority in a paternalistic sense. Explicit criticism of medical authority was new, as it was in society more generally. The emphasis on independent diabetics saw major continuities with the 1950s, but independence accrued personal rather than social meaning. The emphasis was put on unique individuals who needed to experience and get to know their own personal diabetes. Managing one’s disease in order to be healthy was considered to be a process that was dependent on unique personal characteristics and experiences in combination with medical expertise.

As such, the tension between discipline and independence gradually dissolved, and the need to balance the two became less important. As a consequence, the idea of a ‘good diabetic’ as a ‘model citizen’ got less prominence too. Organised diabetics did renew the emphasis on diabetic health in the late 1960s, but this was different from the 1950s social concept of health with its strong moral connotations with functioning as a ‘normal member’ of society in family, labour and social life. In the new personal concept of health, organised diabetics regarded personal experiences with diabetes as an essential source of knowledge. As a culmination of these changes, the Dutch Diabetics Association replaced the word ‘*suikerzieken’* with ‘diabetes’ in its name in 1972.

This study of diabetes discourse within the Dutch Diabetics Association shows that patient autonomy that historians have generally located in the late 1960s and 1970s, had at that point changed in meaning.^156^ From being socially independent by following medical rules prescribed by medical authorities, patient autonomy had come to mean making medical decisions and be heard as experts, involving collaboration between healthcare professionals and patients. Compared to North American and British diabetes associations led by doctors, and even the more similar German and New Zealand associations led by medical doctors and diabetics,^157^ Dutch diabetics had a relatively active role on national, local and international levels of organisation, and explicitly linked this role to the aim of independence. Attuned to the atmosphere of post-war reconstruction with sense of duty as a central societal value, this was patient autonomy in a social sense, not the more individualised patient autonomy of later decades. Moore’s argument that ‘medicine’s expanded management strategies had limits’^158^ is therefore true for the Dutch case in particular, also because the Netherlands lacked a leading medical diabetes specialist.

The major explanation for this is the Dutch ‘patchwork’ healthcare system in which the post-war expansion of the welfare state was combined with a more general ideal of citizens’ organisation and independence. The many NVS education activities on local and national levels involved diabetics, an eclectic field of healthcare professionals including medical doctors, nurses and government dieticians, but also representatives of civil organisations, insurance companies etcetera. It is clear that this collaborative education played a crucial role in the discourse on diabetic health. What both Moore and Harper analyse as new interest in patient education and self-management in British and New Zealand diabetes care of the 1970s and 1980s, was not so new in the Netherlands as a consequence. What these new insights in the histories of diabetes organisations mean for our understanding of broader patient movements and patient consumerism in the second half of the twentieth century,^159^ is an important question for further research.

Understanding changes in disease concepts is important, because they have effects.^160^ The shifts in discourse I have analysed, have likely influenced the life and work of different social groups of diabetes patients and their caretakers. However, I have only superficially touched upon the question what such effects were. We need for example more insight what the NVS’ diabetes education meant for diabetics and healthcare professionals in a practical sense. Paying attention to differences in class, ethnicity, gender, age and profession (general practitioners, medical specialists, dieticians, nurses) is particularly important here, because ideas about health, balance and disease were so value-laden. How did the focus on independence and social health affect patients’ lives and healthcare professionals’ work? What changed when a more personal understanding of living healthy with diabetes arose? And what did this mean for patients who did not succeed in reaching balance and health?

## Footnotes

2 ‘Het tien-jarig Jubileum’, *Het Tijdschrift van de Nederlandse Vereniging van Suikerzieken*, 1955, *9*, 158–68, 159–60. The author has translated all Dutch quotations to English.

3 Martin D. Moore, ‘Balance and the “Good” Diabetic in Britain, C.1900–60’, in Mark Jackson and Martin D. Moore, eds, *Balancing the Self: Medicine, Politics and the Regulation of Health in the Twentieth Century* (Manchester: Manchester University Press, 2020), 33–63.

4
*Ibid.*, 34.

5 John Christopher Feudtner, *Bittersweet: Diabetes, Insulin, and the Transformation of Illness* (Chapel Hill: University of North Carolina Press, 2003).

6 Martin D. Moore, *Managing Diabetes, Managing Medicine: Chronic Disease and Clinical Bureaucracy in Post-War Britain* (Manchester: Manchester University Press, 2019), 18.

7 Arleen Tuchman, *Diabetes: A History of Race and Disease* (New Haven: Yale University Press, 2020), 63.

8
*Ibid.*, 32–68; Chris Feudtner, ‘The Want of Control: Innovations and Ideals in the Modern Management of Diabetes’, *Bulletin of the History of Medicine*, 1995, 69, 66–90; Moore, ‘Balance’.

9 John Christopher Feudtner, *Bittersweet: Diabetes, Insulin, and the Transformation of Illness* (Chapel Hill: University of North Carolina Press, 2003); Oliver Falk, ‘Der Patient als epistemische Größe: Praktisches Wissen und Selbsttechniken in der. Diabetestherapie 1922-1960’, *Medizinhistorisches Journal*, 2018, 53, 36–58.

10 Martin D. Moore, ‘Food as Medicine: Diet, Diabetes Management, and the Patient in Twentieth Century Britain’, *Journal of the History of Medicine and Allied Sciences,* 2018, 73, 150–67.

11 Moore, ‘Balance’.

12 Tuchman, *Diabetes.*

13 Feudtner, *Bittersweet,* ch. 7; C.R. Prüll, ‘Making Sense of Diabetes: Public Discussions in Early West Germany 1945 to 1970’, in Jörg Rogge, ed., *Making Sense as a Cultural Practice: Historical Perspectives* (Bielefeld: 2013), 225–39; Tuchman, *Diabetes,* ch. 2.

14 G. Krzentowski, ‘Le rôle des associations du diabète’, *Revue Medicale de Liège*, 2005, 60, 612–614; C. R. Prüll, ‘Auf der Suche nach dem “Zucker-Mädchen” - Sexualität und Partnerschaft im Journal “Der Diabetiker” (1951–1970)’, *Medizinhistorisches Journal*, 2012, 47, 31–61; Courtney E. Harper, ‘“Towards Self-Sufficiency”: Public Health Responses to Diabetes in New Zealand C.1920–2000’ (unpublished PhD thesis, University of Auckland, 2015), ch. 2.

15 Moore, ‘Balance’.

16 Prüll, ‘Auf der Suche’; C.R. Prüll, ‘Making Sense of Diabetes: Public Discussions in Early West Germany 1945 to 1970’, in Jörg Rogge, ed, *Making Sense as a Cultural Practice: Historical Perspectives* (Bielefeld: 2013); Harper, ‘“Towards Self-Sufficiency”’, ch. 2.

17 Nancy Tomes and Beatrix Hoffman, ‘Introduction’, in Beatrix Hoffman *et al*., eds, *Patients as Policy Actors: A Century of Changing Markets and Missions* (New Brunswick: Rutgers University Press, 2011) 1–16, 12; Steven Epstein, ‘Patient Groups and Health Movements’, in Edward J. Hackett *et al.*, eds, *The Handbook of Science and Technology Studies* (Cambridge USA: MIT Press, 2007), 499–539; Alex Mold, *Making the Patient-Consumer: Patient Organisations and Health Consumerism in Britain* (Manchester: Manchester University Press, 2015) 1–17.

18 J. C. Burnham, ‘The Death of the Sick Role’, *Social History of Medicine*, 2012, 25, 761–76; H. Huistra, ‘Experts by Experience: Lay Users as Authorities in Slimming Remedy Advertisements, 1918–1939’, *BMGN—Low Countries Historical Review,* 2017, 132, 126–48.

19
*Het Tijdschrift van de Nederlandsche Vereeniging tot Behartiging van de Belangen van Suikerzieken* (1946–1947); *Het Tijdschrift van de Nederlandse Vereniging van Suikerzieken* (1948–1956); *Het Tijdschrift* (1957–1965); *NVS Nieuws* (1966–1970); *Diabc* (1970).

20 ‘Een boodschap van Ir. A. Mollinger’, *T. NVS*, 1957, 11, 173–74.

21 Jeremy A. Greene, *Prescribing by Numbers: Drugs and the Definition of Disease* (Baltimore: Johns Hopkins University Press, 2007), 87–88; Moore, *Managing,* 21.

22
*Ibid*. (quote); Feudtner, *Bittersweet*; Moore, ‘Balance’.

23 Van Klaveren, *Het onafhankelijkheidssyndroom.*

24 Harper, ‘“Towards Self-Sufficiency”’, 86–87.

25 Moore, *Managing*, 21; S. Lissauer, ‘De zorg voor de Suikerpatient in Engeland’, *T. NVS*, 1950, 4:3, 13–15; S. Lissauer, ‘Het tweede Internationale Diabetes-Congres te Cambridge’, *T. NVS*, 1955, 9, 84–87.

26 J.A. Martijn, ‘Bezinning’, *T. NVS*, 1955, 9, 135–7, 135.

27 The founding name *Nederlandsche Vereeniging tot Behartiging van de Belangen van Suikerzieken* was changed into *Nederlandse Vereniging van Suikerzieken* in 1948.

28 In March 1951, the NVS had 47 local departments and 11,464 members. At the end of 1967, the NVS had 70 local departments and 27,476 members. J.D. Boks, ‘Overzicht ledental der N.V.S.’, *T. NVS,* 1951, 5:2, 2; ‘Verslag van het Hoofdbestuur over het verenigingsjaar 1967’, *T. NVS,* 1968, 22, 103–17, 111–12.

29 Van Klaveren, *Het onafhankelijkheidssyndroom,* ch. 1 and 2; Piet de Rooy, *A Tiny Spot on the Earth: The Political Culture of the Netherlands in the Nineteenth and Twentieth Centuries* (Amsterdam: Amsterdam University Press, 2015), ch. 6.

30 Nederlandse Vereniging van Suikerzieken, ‘Statuten en huishoudelijk reglement’, 1947, inv. nr. 625, BNR 277-01, Stichting Sociale Raad, Gemeentearchief Den Haag.

31 Prüll, ‘Making Sense’, 229–30.

32 At the start, the editorial board consisted of two medical doctors (including the medical advisor) and one lay NVS board member. From December 1947 until 1952, the editorial board consisted of two medical doctors, one dietician and one lay NVS board member. From 1953 until 1963, the editorial board consisted of two medical doctors and two lay NVS board members. From 1963 onwards, lay members were the majority on the editorial board.

33 H.S. Roosje, ‘1949-1950’, *T. NVS*, 1949, 3:6, 2–3.

34 A.J. Mollinger, ‘Tien jaar N.V.S.’, *T. NVS*, 1955, 9, 115–6.

35 F. Gerritzen, ‘Bij het 10-jarig bestaan der N.V.S.’, *T. NVS*, 1955, 9, 122–3.

36 E.g.: S. Lissauer, ‘Een bespreking met het Bestuur van de I.D.F.’, *T. NVS*, 1956, 10, 16.

37 Roosje, ‘1949–1950’.

38 R.D. Lawrence and F. Gerritzen, ‘De Internationale Diabetes Federatie (I.D.F.)’, *T. NVS*, 1951, 5:4, 2–3; S. Lissauer, ‘Uit de Internationale Diabetes Federatie (I.D.F.)’, *T. NVS*, 1955, 9, 58–60.

39 Geert Somsen, ‘“Holland’s Calling”: Dutch Scientists as International Mediators in the Interwar Period’, in Sven Widmalm, Geert Somsen, Rebecka Lettevall, eds, *Neutrality: Science, Culture and Politics after the First World War* (New York and London: Routledge, 2012), 45–64.

40 Lissauer, ‘Uit de Internationale Diabetes Federatie’, 1955, 58; Lissauer, ‘Het tweede Internationale Diabetes-Congres’.

41 F. Gerritzen, ‘Het Congres van de I.D.F.’, *T. NVS*, 1952, 6:4, 2–5.

42 The NVS was represented in the IDF Board by its medical advisor, and in the IDF Council by the chairman of its Medical Advisory Board and the NVS Board president.

43 Lawrence and Gerritzen, ‘De Internationale Diabetes Federatie’, 3.

44 C. den Hartog, ‘Wat denken onze medici’, *T. NVS*, 1948, 2:2, 9–10; F. Gerritzen, ‘Goede raadgevingen’, *T. NVS*, 1954, 8:1, 3–4.

45 Of the 14 founding members of the Medical Advisory Board of the NVS, two members were affiliated to the government Dutch Nutrition Education Bureau (director dr. C. den Hartog and dietician D.J. ten Haaf), and one member was a nurse (H. Th. M. Dröge). J.D. Boks, ‘Van de Secretaris’, *Het Tijdschrift van de Nederlandsche Vereeniging tot Behartiging van de Belangen van Suikerzieken*, 1947, 1:4, 2–6, 2. See on the Dutch Nutrition Education Bureau: J. Verriet, ‘Struggling Over Healthy Lifestyles: The Dutch Nutrition Education Bureau and the Individualisation of Public Health (1940-1980)’, *BMGN—Low Countries Historical Review,* 2021, 136, 4–32.

46 E.g.: ‘De Gewestelijke Bijeenkomst te Amsterdam’, *T. NVS*, 1952, 6:5, 12–4; B. Wartena, ‘Maatschappelijke Diabeteszorg’, *T. NVS*, 1950, 4:1, 8–12; F. Gerritzen, ‘De plaats der diabetici in de samenleving’, *T. NVS*, 1955, 9, 90–93.

47 Gerritzen, ‘De plaats’, 90; S. Lissauer, ‘Een goedgeslaagde Jongeren-bijeenkomst’, *T. NVS*, 1956, 10, 19–20.

48 F. Gerritzen, ‘Complicaties van diabetes’, *T. NVS*, 1951, 5:4, 13.

49 F. Gerritzen, ‘Suikerziekte bij zwaarlijvige diabeten’, *T. NVS*, 1952, 6:1, 6–7.

50 S.L., ‘Een praatje over voeten.’, *T. NVS*, 1949, 3:2, 15–17, 15.

51 ‘U vraagt, wij antwoorden’, *T. Ned. Ver. Beh. Belang. Suikerz.*, 1947, 1:2, 5–7, 6.

52 Feudtner, *Bittersweet*; Tuchman, *Diabetes*; Falk, ‘Der Patient’; Prüll, ‘Making Sense’; Moore, ‘Balance’.

53 De Rooy, *A Tiny Spot,* 241.

54 Van Klaveren, *Het onafhankelijkheidssyndroom.*

55 Feudtner, *Bittersweet*, ch. 5; Prüll, ‘Auf der Suche’, 38–39.

56 E.g.: J.J.C. Mantz, ‘De samenstelling van de voeding’, *T. NVS*, 1954, 8:1, 13–14; Th. Dankelman, ‘De buitendienst van de Diabetes-Nazorg van het Centrum Heerlen’, *T. NVS*, 1954, 8:1, 11–12; F.S.P. van Buchem, ‘Het 10-jarig bestaan van de N.V.S.’, *T. NVS*, 1955, 9, 114.

57 N. Bloemendaal, ‘Jubileum-Kroniek’, *T. NVS*, 1955, 9, 126–33, 128.

58 Lissauer, ‘Het tweede Internationale Diabetes-Congres’, 85.

59 Van Klaveren, *Het onafhankelijkheidssyndroom,* ch. 1 and 2; D. Bos, F. Wekker, and J.W. Duyvendak, ‘Thuis best: Over het Nederlandse ideaal van zorg met behoud van zelfstandigheid’, in T. Kampen, I. Verhoeven, and L. Verplanke, eds, *De affectieve burger: Hoe de overheid verleidt en verplicht tot zorgzaamheid* (Amsterdam: Van Gennep, 2013), 133–53.

60 S. Lissauer, ‘Tien jaren “selfhelp”’, *T. NVS*, 1955, 9, 121–2.

61 R.D. Lawrence, ‘Goede gezondheid door juist evenwicht’, *T. NVS*, 1955, 9, 52–54.

62 S. Lissauer, ‘De suikerzieke en de arbeidsmarkt’, *T. NVS*, 1956, 10, 99.

63 J.A. Martijn, ‘Bezinning’, *T. NVS*, 1955, 9, 135–7, 137.

64 Lissauer, ‘Een goedgeslaagde Jongeren-bijeenkomst’.

65 Gerritzen, ‘De plaats’, 90; ‘Verslag van een gesprek tussen Dr. J. Groen en vijf suikerzieken’, *T. NVS*, 1957, 11, 41–44; S. Lissauer, ‘Uit de diabetische wereld’, *T. NVS*, 1958, 12, 11–14; ‘Afdelingsnieuws’, *T. NVS*, 1958, 12, 186–9, 187.

66 ‘Verslag van een gesprek’, 42.

67 J.J. Witte, ‘Het kindertehuis “Bos en Duin” te Huis ter Heide’, *T. NVS*, 1961, 15, 177–80, 178.

68 The last sentence in Dutch reads: ‘Tot heil van onze, niet ziek zijnde, suikerzieke kinderen!’ L. Andersson, ‘Klaar? Af!’, *T. NVS*, 1959, 13, 141–3.

69 Lissauer, ‘Tien jaren “selfhelp”’.

70 Moore, *Managing*, ch. 1.

71 ‘Het Internationaal Congres te Cambridge’, *T. NVS*, 1955, 9, 62.

72 Nelleke Bakker, ‘Sunshine as Medicine: Health Colonies and the Medicalization of Childhood in the Netherlands C.1900-1960’, *History of Education*, 2007, 36, 659–679.

73 ‘Wij gaan uit … en doen gewoon: Onze nieuwe film’, *T. NVS*, 1955, 9, 175.

74 Greet Edema, ‘Wij gingen uit, omdat wij gewoon zijn’, *T. NVS,* 1957, 11, 141–3; ‘Reünie te “Bos en Duin”’, *T. NVS,* 1959, 13, 170–171. Compare Moore, ‘Balance’, 48.

75 M.J.H.F. Wevers, ‘Consuming America: A Data-Driven Analysis of the United States as a Reference Culture in Dutch Public Discourse on Consumer Goods, 1890–1990’ (unpublished PhD thesis, Utrecht University, 2017).

76 Feudtner, *Bittersweet*, ch. 7.

77 ‘Van de afdelingen’, *T. NVS*, 1954, 8:2, 19–23, 22–23.

78 S. Lissauer, ‘De zorg voor de Suikerpatient in Engeland’, *T. NVS*, 1950, 4:2, 17–18.

79 Compare Jolanda Dwarswaard and Margo Trappenburg, ‘De huisarts en de tijdgeest’, *Huisarts en wetenschap*, 2012, 55, 504–8.

80 ‘Antwoorden op belangrijke vragen’, *T. NVS*, 1956, 10, 141–6, 146.

81 ‘Levensverzekering voor Suikerzieken’, *T. NVS*, 1954, 8:5, 4–6, 4.

82 S. Lissauer, ‘De zorg voor de Suikerpatient in Engeland’, *T. NVS*, 1950, 4:6, 21–22.

83 Christiane Sinding, ‘The Specificity of Medical Facts: The Case of Diabetology’, *Studies in History and Philosophy of Biology & Biomedical Sciences*, 2004, 35, 545–59; Moore, ‘Balance’, 38.

84 F.G. Schlesinger, ‘Medische gegevens van het 3e I.D.F.-Kongres (slot)’, *T. NVS*, 1959, 13, 6–8, 7.

85 Diane J. ten Haaf, ‘Van de diëtiste’, *T. Ned. Ver. Beh. Belang. Suikerz.*, 1947, 1:2, 2–4, 2.

86 The Dutch word *voorschrift* was elsewhere explicitly discussed as meaning a strict prescription: H.S. Roosje, ‘Van de Voorzitter’, *T. NVS*, 1948, 2:4, 3.

87 Ten Haaf, ‘Van de diëtiste’.

88 J.J.C. Mantz and C. den Hartog, ‘Het moderne diabetes-dieet’, *T. NVS*, 1960, 14, 105–6.

89 Bloemendaal, ‘Jubileum-Kroniek’, 126.

90 Lissauer, ‘Een goedgeslaagde Jongeren-bijeenkomst’. Parents, poor parents in particular, were more generally considered a bad influence on children in health colonies, compare: Bakker, ‘Sunshine’.

91 Lissauer, ‘Een goedgeslaagde Jongeren-bijeenkomst’.

92 F. Gerritzen, ‘Lastige diabetes-patiënten’, *T. NVS*, 1950, 4:2, 8.

93 M.N. E., ‘Een verschrikkelijke les’, *T. NVS*, 1951, 5:4, 7–8.

94 Lissauer, ‘Een goedgeslaagde Jongeren-bijeenkomst’.

95 Gerritzen, ‘Lastige diabetes-patiënten’.

96 Tuchman, *Diabetes,* ch. 2.

97 Like was the case in the US and Germany: Prüll, ‘Making Sense’; Feudtner, *Bittersweet,* ch. 7.

98 B.E. Rutgers-Mees, ‘Een voorbeeld’, *T. NVS*, 1961, 15, 161.

99 Moore, ‘Balance’, 44; Tuchman, *Diabetes*.

100 ‘Van de Afdelingen’, *T. NVS*, 1956, 10, 148–50.

101 S. Lissauer, ‘Uit de Internationale Diabetes Federatie’, *T. NVS*, 1954, 8:6, 13–15.

102 E.g. ‘Nog een voorbeeld’, *T. NVS*, 1961, 15, 163.

103 Mold, *Making*, 10.

104 Greene, *Prescribing*.

105 C. Schreuder, ‘Diabetes, tabletten en infectie (I)’, *NVS-Nieuws*, 1970, 24, 4–6.

106 To illustrate the growth: during the 1952 IDF congress in Leiden, 150 medical doctors and 100 lay participants were present; while 3500 participants joined the 1964 IDF congress in Toronto, and 2000 (‘mostly doctors’) the 1967 IDF congress in Stockholm. Jac. J. Witte, ‘De Internationale Diabetes Federatie’, *NVS-Nieuws*, 1967, *21,* 38–39, 38; B. Wilton, ‘Het 6e I.D.F.-kongres in Stockholm’, *NVS-Nieuws*, 1967, 21, 119–21.

107 N. Bloemendaal, ‘Diabetes is een gewichtige aangelegenheid’, *T. NVS*, 1961, 15, 115–9, 118. Compare for example the theme issue on labour and choice of profession: *T. NVS*, 1960, 14:5, 85–92.

108 Howard F. Root, ‘Het 5^e^ Kongres van de Internationale Diabetes Federatie te Toronto’, *T. NVS*, 1965, 19, 5–8.

109 E.g. Jac. J. Witte, ‘Van uw Medisch Adviseur’, *T. NVS*, 1961, 15, 67.

110 Jac. J. Witte, ‘Ervaringen met suikerzieke kinderen’, *T. NVS*, 1965, 19, 19–22.

111 In the 1950s, psychoanalysts and educationists argued that taking children from their families and from school was harmful and that the health colonies did not cure mental problems. Bakker, ‘Sunshine’.

112 Jac. J. Witte, ‘De bejaarde suikerzieke’, *T. NVS*, 1965, *19*, 35–37, 35–36; Jac. J. Witte, ‘Toekomst en perspectief!’, *T. NVS*, 1965, *19*, 145–148, 146.

113
*Ibid.,* 147.

114 Jac. J. Witte, ‘Suikerzieken en maatschappij’, *NVS-Nieuws*, 1967, 21, 122–5, 124.

115 Schreuder, ‘Diabetes’, 5.

116 Jac. J. Witte, ‘Van uw Medisch Adviseur’, *T. NVS*, 1961, 15, 35–36, 36.

117 Geert Buelens, *De jaren zestig: Een cultuurgeschiedenis* (Amsterdam: Ambo/Anthos, 2018); De Rooy, *A Tiny* Spot, ch. 7.

118 James Kennedy, *Nieuw Babylon in aanbouw: Nederland in de jaren zestig* (Amsterdam: Boom, 1995).

119 Mold, *Making*, 1–17.

120 Several local departments criticised the daily board for not communicating open enough with the association. Unfortunately, the journal of this new Amsterdam association, *Dia’m: Uitgave van De Nederlandse Diabetes Stichting Amsterdam* (1967–1969) is not preserved: the copies of the University of Amsterdam Library are lost, and the Diabetesvereniging Nederland does not have copies either.

121 Th. R. Janze, ‘Om de S van de N.V.S.’, *NVS-Nieuws*, 1966, 20, 55–56; J. Delcour and Th. R. Janze, ‘Nogmaals S’, *NVS-Nieuws*, 1966, 20, 110; K.W. de Langen, ‘Suikerzieken of diabetici?’, *NVS-Nieuws*, 1967, 21, 181.

122 P. Vermaire-Vader, ‘Afd. Walcheren en omstr.’, *NVS-Nieuws*, 1968, 22, 74; ‘Meer brieven over naam voor tijdschrift’, *NVS-Nieuws*, 1969, 23, 44; ‘NVS Diabrief’, *Diabc,* 1970, 24, 146–7.

123 ‘Reacties op het weekend in Kortehemmen’, *Diabc,* 1970, 24, 197.

124 A.F. Burmeister-Visser, ‘Ervaringen van een gezonde suikerzieke’, *NVS-Nieuws,* 1968, 22, 87–88.

125 For a more extensive discussion of the rise of personal experience as an influential form of expertise in the late 1960s, see, for example: Jennifer Crane, *Child Protection in England, 1960–2000: Expertise, Experience, and Emotion* (Palgrave Macmillan, 2018).

126 Van Klaveren, *Het onafhankelijkheidssyndroom,* 25.

127 Jan Koehaar, ‘Dat zijn mijn belevenissen’, *T. NVS*, 1962, 16, 144–5.

128 ‘Afdelingsnieuws’, *NVS-Nieuws,* 1968, *22*, 133–4, 133.

129 A. Burmeister-Visser, ‘Ervaringen van een gezonde suikerzieke’, *Diabc*, 1970, 24, 65.

130 M. de Jong, ‘Voorwoord’, *Diabc*, 1970, 24, 55.

131 P. Verhage, ‘Hoe ik een hypoglycaemie des nachts kan voorkomen’, *Diabc*, 1970, 24, 61–62.

132 ‘NVS Diabrief’, *Diabc*, 1970, 24, 146–7.

133 ‘Reacties op het weekend’.

134 E.g.: Koehaar, ‘Dat zijn mijn belevenissen’; M.N. Kiesouw, ‘Toelichting op Omgeving en Suikerziekte’, *T. NVS*, 1965, 19, 60–61; ‘Achteraf…’, *NVS-Nieuws*, 1967, 21, 54; ‘Echt gebeurd!’, *NVS-Nieuws*, 1968, 22, 150; ‘NVS diabrief’, *Diabc*, 1970, 24, 185.

135 Prüll, ‘Auf der Suche’; Dwarswaard and Trappenburg, ‘De huisarts’; Stuart Bradwel, ‘“Doctor’s Orders”: Type 1 Diabetes and the Consultative Relationship, 1948–2002’ (unpublished PhD thesis, University of Strathclyde, Glasgow, 2020), 50–51.

136 ‘Afdelingsnieuws’, *NVS-Nieuws,* 1968, 22, 133–4, 133.

137 ‘NVS diabrief’, *Diabc*, 1970, 24, 146–147.

138 Jac. J. Witte, ‘Bevolkingsonderzoek op suikerziekte’, 1968, 22, 171–2.

139 ‘Algemene ledenvergadering te Utrecht’, *NVS-Nieuws*, 1969, 23, 168, 170.

140 ‘Afdelingsnieuws’, *NVS-Nieuws,* 1968, 22, 133–4, 133.

141 ‘Verenigingsnieuws’, *Diabc*, 1970, 24, 188.

142 Moore, ‘Balance’, 48.

143 The NVS journal was simply renamed ‘The Journal’ (*Het Tijdschrift*) in 1957, ‘NVS News’ (*NVS-Nieuws*) in 1965, and *Diabc* in 1970.

144 Jac. J. Witte, ‘NVS moet doorgroeien naar volwassenheid’, *NVS-Nieuws,* 1968, 22, 140.

145 Kathrin Hiepko, ‘“Conditionally Healthy and Able to Work”: Diabetes Prevention, Care and Research in the German Democratic Republic (GDR), c.1949–1990’ (unpublished PhD thesis, University of Manchester, 2019).

146 E.g.: *Ibid.;* Jac. J. de Jong, ‘Suikerziekte en huwelijk’, *T. NVS*, 1965, 19, 103; Jac. J. Witte, ‘Suiker en suikerziekte’, *NVS-Nieuws,* 1968, 22, 3–5; Jac. J. Witte, ‘Suikerziekte en Rijvaardigheidsbewijzen (vervolg en slot)’, *NVS-Nieuws,* 1968, 22, 35–36.

147 M.C. Reckman, ‘Ir. B. Wilton: NVS moet snelwerkend instituut worden’, *NVS-Nieuws,* 1968, 22, 173.

148 ‘Dr. Jac. J. Witte verlaat NVS’, *NVS-Nieuws,* 1969, 23, 19; ‘Ir. B. Wilton legt het voorzitterschap van de NVS neer’, *NVS-Nieuws*, 1969, 23, 39.

149 M. de Jong and J. Rotte, ‘De nieuwe Medisch Adviescommissie’, *Diabc*, 1970, 24, 120.

150 Director of the Food Education Bureau and medical doctor Den Hartog left the Medical Advisory Board and the editorial board of the NVS journal too in March 1969.

151 De Jong and Rotte, ‘De nieuwe Medisch Adviescommissie’.

152 The NVS ‘structure committee’ was appointed by the general board after the Amsterdam department left the NVS in the autumn of 1968. Its chairman, notary Z. Deenik played an important role in solving this conflict, leading to the return of the Amsterdam department in the NVS in the summer of 1969. The committee wrote a report about changes in the formal structure of the NVS, for example to incorporate the separate Amsterdam Dutch Diabetics Foundation in the NVS, in consultation with the local departments and the board. From April 1970 onwards, it focussed on the major issue of renaming the NVS. ‘Buitengewone algemene ledenvergadering van de afdeling Amsterdam e.o.’, *NVS-Nieuws,* 1969, 23, 143–4; Z. Deenik, ‘Hoe gaan wij onze vereniging noemen?’, *Diabc,* 1970, *24*, 59.

153 ‘Verenigingsnieuws’, *Diabc,* 1970, 24, 127–9, 127.

154 Moore, ‘Balance’.

155
*Ibid.*

156 Compare Mold, *Making*, ch. 1; Moore, ‘Food’, 153; Harper, ‘“Towards Self-Sufficiency”’, ch. 4.

157 Compare Greene, *Prescribing,* 87–88; Moore, *Managing,* 15; Prüll, ‘Making Sense’; Harper, ‘“Towards Self-Sufficiency”’.

158 Moore, ‘Food’, 152.

159 Mold, *Making.*

160 Robert Aronowitz, ‘Framing Disease: An Underappreciated Mechanism for the Social Patterning of Health’, *Social Science and Medicine*, 2008, 67, 1–9.

## Notes

Floor Haalboom is an assistant professor of medical history at Erasmus University Medical Center Rotterdam. She writes and teaches about the modern history of disease and the environmental history of livestock farming. This article resulted from the project ‘Health and disease as practical concepts: a pragmatist approach to conceptualisation of health and disease’. Haalbooms current research project is called ‘What does your meat eat? A global environmental history of Dutch livestock feed (1954–2020)’.

